# Elucidation of sex from mature Palmer amaranth (*Amaranthus palmeri*) leaves using a portable Raman spectrometer†

**DOI:** 10.1039/d3ra06368b

**Published:** 2024-01-08

**Authors:** Aidan P. Holman, Nicolas K. Goff, Isaac D. Juárez, Samantha Higgins, Axell Rodriguez, Muthukumar Bagavathiannan, Dmitry Kurouski, Nithya Subramanian

**Affiliations:** a Department of Entomology, Texas A&M University College Station Texas 77843 USA; b Department of Biochemistry and Biophysics, Texas A&M University College Station Texas 77843 USA dkurouksi@tamu.edu; c The University of Texas at Austin Dell Medical School Austin Texas 78712 USA; d Institute for Advancing Health Through Agriculture College Station Texas 77843 USA; e Department of Soil and Crop Sciences, Texas A&M University College Station Texas 77843 USA muthu@tamu.edu nithya.subramanian@ag.tamu.edu

## Abstract

Palmer amaranth (*Amaranthus palmeri*) is a pervasive and troublesome weed species that poses significant challenges to agriculture in the United States. Identifying the sex of Palmer amaranth plants is crucial for developing tailored control measures due to the distinct characteristics and reproductive strategies exhibited by male and female plants. Traditional methods for sex determination are expensive and time-consuming, but recent advancements in spectroscopic techniques offer new possibilities. This study explores the potential of portable Raman spectroscopy for determining the sex of mature Palmer amaranth plants in-field. Raman analysis of the plant leaves reveals spectral differences associated with nitrate salts, lipids, carotenoids, and terpenoids, allowing for high accuracy and reliable identification of the plant's sex; male plants had higher concentrations of these compounds compared to females. It was also found that male plants had higher concentrations of these compounds compared to the females. Raman spectra were analyzed using a machine learning tool, partial least squares discriminant analysis (PLS-DA), to generate accuracies of no less than 83.7% when elucidating sex from acquired spectra. These findings provide insights into the sex-specific characteristics of Palmer amaranth and suggest that Raman analysis, combined with PLS-DA, can be a promising, non-destructive, and efficient method for sex determination in field settings. This approach has implications for developing sex-specific management strategies to monitor and control this invasive weed in real-world environments, benefiting farmers, agronomists, researchers, and master gardeners.

## Abstract 2

Palmer amaranth (*Amaranthus palmeri*) is a pervasive and troublesome weed species that poses significant challenges to agriculture in the United States.

## Graphical abstract

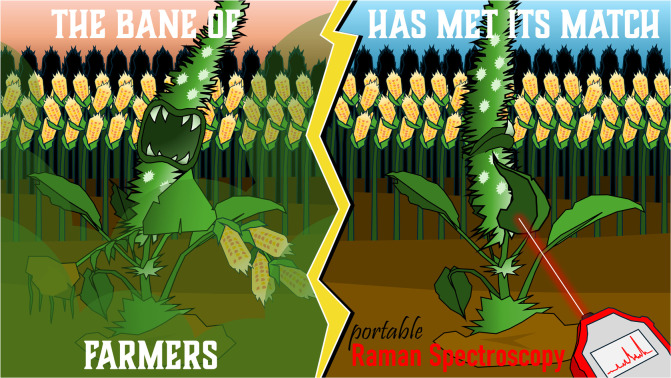

## Introduction

Palmer amaranth (*Amaranthus palmeri* S. Wats.) is an aggressive and highly problematic weed species that poses significant challenges to agricultural practices across various regions of the United States.^1^ Native to the southwestern United States, Palmer amaranth has rapidly spread to other parts of the country, infesting croplands, pastures, and even natural habitats. Its adaptability, prolific seed production, and resistance to multiple herbicides make it an insidious threat to agricultural productivity and sustainability.

Interestingly, the ability to discern the sex of Palmer amaranth plants plays a pivotal role in developing tailored control measures. Palmer amaranth is a dioecious species, with separate male and female plants, making their identification vital for implementing sex-specific management approaches. Female plants can be especially targeted in order to reduce seed production, minimize seedbank replenishment, and curb long-term population growth and species persistence.^2^

Current methods for the determination of Palmer amaranth sex rely on visual inspections of morphological characteristics requiring extensive training^3^ or the use of genetic markers,^4^ which is expensive and time-consuming. However, the recent advancements in spectroscopic techniques have shown immense potential in revolutionizing agricultural practices. Among these techniques, Raman spectroscopy (RS) stands out as a promising tool for plant analysis due to its non-destructive nature, rapid data acquisition, and ability to identify molecular constituents. RS utilizes the interaction between light and organic matter to provide valuable insights into the molecular composition of a sample. By measuring the scattered light, it can reveal detailed molecular fingerprint information, allowing for the characterization and discrimination of various compounds. In the context of agriculture, RS has been successfully applied in diverse areas such as crop disease diagnosis,^5^ nutrient analysis,^6^ pesticide residue detection,^7,8^ and quality assessment of agricultural products.^9^ Of present concern, Higgins *et al.* (2022) used a portable Raman spectrometer to accurately identify the sex of mature hemp plants by scanning their leaves.^10^

In this study, we aim to explore the potential of portable Raman spectroscopy for elucidating the sex of mature Palmer amaranth plants. The development of a rapid and accurate method for sex determination using a handheld Raman spectrometer would enable farmers, agronomists, and researchers to efficiently identify the sex of Palmer amaranth plants in the field, facilitating the implementation of targeted management strategies.

## Materials and methods

### Plant treatment

A Palmer amaranth (hereafter Palmer) population collected from Lubbock County, TX was used in this study. The study was repeated twice during summer 2022 and fall 2022. The Palmer seeds were planted in rectangular trays (25 × 50 cm; two batches) filled with Joly Gardener, Pro-line C/20 potting mix in the greenhouse. After germination (approximately 10 days after planting), the seedlings were transplanted into small square pots (8.5 cm depth and 9 cm width) and maintained under greenhouse conditions (32/26 °C day/night, 16 hours of light). Palmer plants were fertilized twice during the experiment and were watered regularly.

Mature Palmer plants of known sex at flowering stage (20 female and 20 male plants) were used for Raman measurements during May 2022 "first run". A model was built using spectral data from these 40 plants. Young leaf tissues from the mature plants at the flowering stage were also collected for HPLC analysis of carotenoids. Following this, Raman spectra were collected for the 40 Palmer plants, at different growth stages from the seedling stage (10–12 cm stage) to heading stage on a weekly interval for 3–4 weeks. Attempts were made to predict the sex of the seedlings and confirm the model using the spectral data from these 40 Palmer plants. The sex of the seedlings was further confirmed visually at plant maturity.

For the second experimental run during October 2022 "second run", two batches of Palmer seedlings were grown in the greenhouse with 14 days interval between the batches. The seeds were first planted in trays and the germinated seedlings were transplanted to small pots after 12 days. Once the transplanted Palmer seedlings were established (two weeks after transplanting), they were separated according to their height. This resulted in three groups each for the first and second batches. Height data (from the top of the soil to the last leaf intersection) for each plant was recorded 14, 19, and 24 days after transplanting for the first batch, and 14, 20, and 26 days after transplanting for the second batch. Raman spectra were also collected on the same day as plant height measurement. The gender of each plant was confirmed when the plants reached the maturity stage. Raman spectra for 35 mature Palmer plants of known sex at flowering stage were also recorded.

### Raman spectroscopy

RS spectra were acquired using a portable, hand-held Raman spectrometer (Agilent Technologies Inc.) with a built-in 830 nm laser. Three spectra were acquired from each leaf at 1 s acquisition time and baseline-subtracted using device software (pre-extraction).

### Carotenoid extraction

Palmer leaf samples, weighing approximately 150 mg, were homogenized using a mortar and pestle. A solution of chloroform and dichloromethane (2 : 1, v/v) with a volume of 1.5 mL was added to the homogenate, which was then agitated on a thermomixer at 500 rpm at 4 °C for 30 minutes. A phase separation was achieved by adding 0.5 mL of 1 M sodium chloride solution to the homogenate and mixing it by inversion. The solution was then centrifuged at 5000 g for 10 minutes, and the aqueous and organic phases were separated into different tubes. The aqueous phase was subjected to another round of separation by adding 0.75 mL of chloroform and dichloromethane (2 : 1, v/v), followed by centrifugation at 5000 g for 10 minutes. The second organic phase was collected and combined with the first batch, and the resulting mixture was dried using a centrifugal evaporation method. The dried pellet was then re-dissolved in 1 mL of methanol/*tert*-butyl ether (MTBE) (60/40, v/v) prior to being injected into the HPLC system.

### HPLC performance

The carotenoids present in leaf extracts were separated and analyzed using a reversed-phase HPLC system equipped with a photodiode array detector (PDA). A reverse-phase C30, 3 mm column was used with a gradient elution method involving mobile phases composed of (A) methanol/water (95 : 5, v/v) and (B) MTBE. The elution profile used for this method began with 97% (A) and 3% (B), which increased linearly to 100% (B) at 20 minutes and then returned to initial conditions at 23 minutes. The column temperature was maintained at 20 °C, and the eluting peaks were monitored at 450 nm using PDA. Quantification of the results was carried out using Breeze software, which involved comparing the peak area with standard reference curves.

### Data analysis

A baseline correction was automatically applied to spectra within the instrument software prior to exportation during acquisition. All spectra were area-normalized before analysis using MATLAB (Mathworks). Chemometric analysis of acquired spectra was done in MATLAB equipped with PLS_Toolbox 9.0 (Eigenvector Research, Inc., Manson, WA). For Partial Least Squares Discriminant Analysis (PLS-DA), 100% calibration-cross validation models were employed; latent variables (LVs) were reported in corresponding tables and chosen lowest root-mean-square error of cross-validation (RMSECV) scores and classification error averages from both calibration and cross-validation, Fig. S1.† The preprocessing for each PLS-DA model, determined through a model optimizer, revealed that the most consistent and highest accuracy was achieved when employing “none,” indicating no preprocessing as the optimal model. Kruskal–Wallis analysis of variance tests (henceforth ANOVA) were utilized to rank specific band height differences between the averaged spectra of each sex.

## Results and discussion

**Fig. 1:**
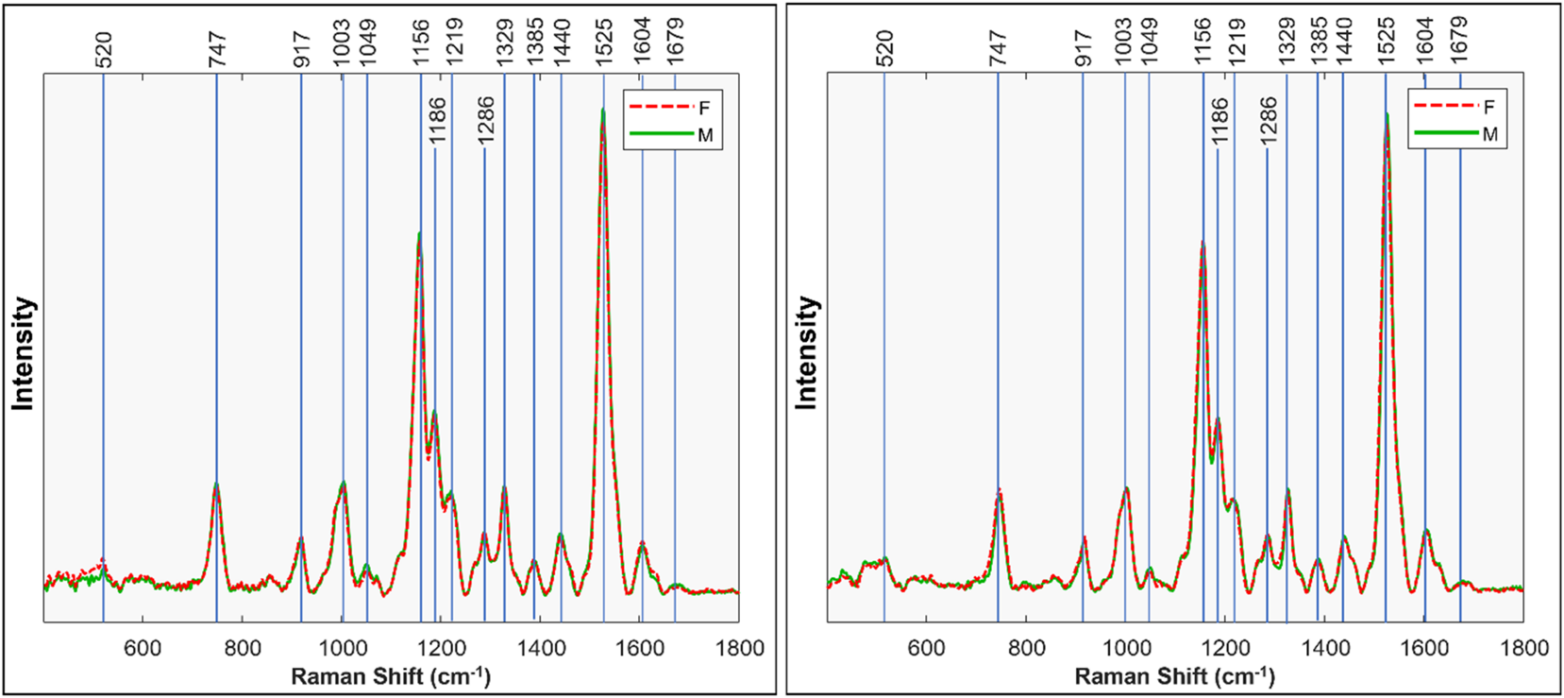
Mean Raman spectra taken from mature Palmer amaranth leaves, classified by sex. Left shows first run, right shows the replicate, second run.

Fig. 1 displays the Raman spectra obtained from male and female Palmer amaranth leaves (mature plants = 40, *n* = 120) (for standard deviations, see Fig. S2†). The spectra show prominent peaks that originate from various biological molecules present in plant tissues.^6,10–12^ Specifically, vibrational bands centered at 520, 747 and 917 cm^−1^ can be assigned to carbohydrates, whereas the peaks at 1156, 1186, and 1525 cm^−1^ originate from polyene vibrations of carotenoids.^11,13^ Carotenoids are pigments that play crucial roles in photosynthesis through the transfer of light, and have antioxidant properties.^14^ We also observed two vibrational bands at 1601 and 1630 cm^−1^, which can be assigned to polyphenols. A peak centered at 1679 cm^−1^ originates from C=C stretching vibration of homoeomorphic rings of terpenoids, the largest group of secondary metabolites in plants that are used to deter predators, inhibit pathogen growth, and protect the plant against oxidative damage caused by environmental stress.^15,16^ In the acquired spectra, we also observe the band at 1049 cm^−1^, which represents the N–O stretching vibration of nitrate.^12^ Plants utilize nitrate salts as a primary source of nitrogen for essential physiological processes such as protein synthesis, enzyme activities, and overall growth and development.^12^ Finally, we find bands at 1329, 1382 and 1440 cm^−1^ that can be assigned to CH and CH_2_ vibrations of lipids.^17^ Lipids are important components of cell membranes and play a crucial role in various physiological processes, including hormone signaling and energy storage.

**Fig. 2:**
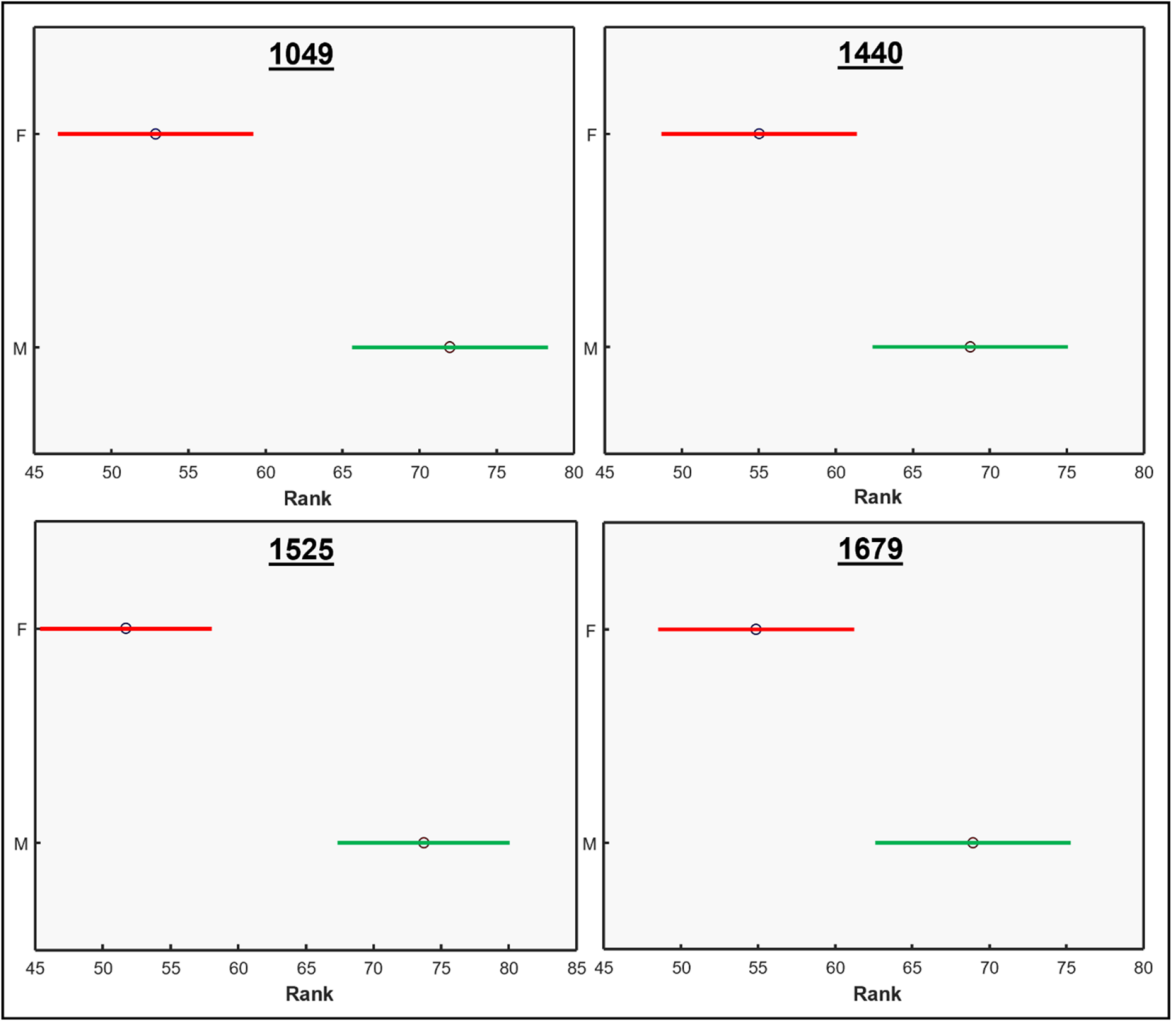
ANOVA tests for bands of physiological importance from the Raman spectra of mature Palmer amaranth leaves, male (green) and female (red). Data displayed is from the first run.

We performed ANOVA to evaluate the statistical significance of differences in relative intensity of 1049 cm^−1^, 1440 cm^−1^, 1525 cm^−1^, and 1679 cm^−1^ bands between the spectra acquired from male and female Palmer amaranth plants, Fig. 2. The results revealed statistically significant differences in intensities of all four vibrational bands (*p* < 0.05). The experiment was repeated (plants = 35, *n* = 105) to ascertain general reproducibility and similar results were obtained, Fig. 1 and S3.†

**Fig. 3:**
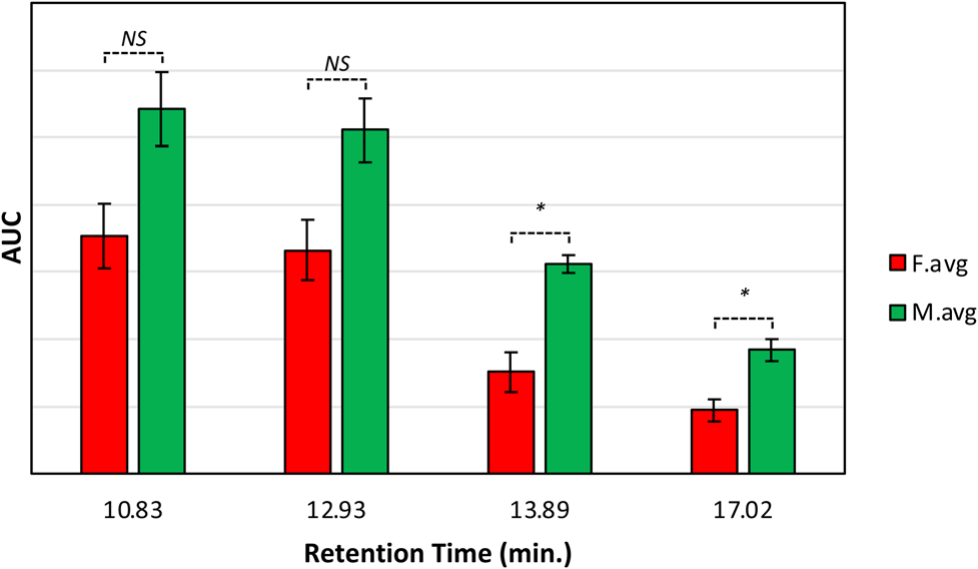
Average peak area and corresponding standard deviations of peaks observed in triplicates of HPLC profiles of mature Palmer amaranth leaves, male (green) and female (red). Retention time (RT) of lutein: 10.83 min, chlorophyll: 12.93 min, pheophytin: 13.89 min, and β-carotene: 17.02 min. Labels indicate *T*-test results: NS is no significance, * is *P* ≤0.05.

These results show that male and female Palmer amaranth plants have statistically significant differences in the concentration of nitrates, carotenoids, lipids, and terpenoids. Specifically, male plants possessed much greater concentrations of these biologically important molecules. To confirm this, we performed an HPLC-based assessment of carotenoids in both male and female Palmer amaranth plants, Fig. 3. HPLC profiles of leaves of Palmer amaranth were dominated by 4 carotenoids: lutein (RT = 10.83 min), chlorophyll (RT = 12.93 min), pheophytin (RT = 13.89 min) and β-carotene (RT = 17.02 min). The HPLC analysis demonstrated that male Palmer amaranth had higher concentrations of all four carotenoids compared to female plants. This difference in concentration was statistically significant for both pheophytin and β-carotene.

We also investigated the potential of Raman analysis to identify the sex of Palmer amaranth through multivariate analysis using PLS-DA. The results showed that the PLS-DA model was able to correctly identify the spectra of male plants in a pool of male and female plants with a high accuracy of 91.7%, Table 1. Additionally, the model demonstrated a relatively high accuracy of 87.5% in correctly identifying spectra from female plants in the same pool. The Matthew's Correlation Coefficient (MCC), a measure of the overall accuracy and reliability of the model, was found to be 0.792, indicating a good discriminatory power. The process was repeated on a different set of plants, and similar results were obtained, with PLS-DA accuracies of 94.6% for males and 83.7% for females, and a MCC of 0.792 again, Table 1. These findings suggest that Raman analysis coupled with PLS-DA has the potential to accurately determine the sex of Palmer amaranth plants, indicating its potential as a reliable and efficient method for sex determination in this invasive weed. The RS could be mounted onto a tractor or drone for large-scale sex determination of Palmer amaranth plants in the field and the implementation of targeted management strategies, especially focusing on late-season female escapes, to reduce viable seed production and seedbank replenishment.

**Table 1:** PLS-DA models for calibration by sex of mature Palmer amaranth leaf spectra. “*n*” refers to the total number of collected spectra per experimental group

1st run		Actual sex		2nd run	Actual sex	
Predicted sex	Accuracy (%)	Female (*n* = 72)	Male (*n* = 48)	Accuracy (%)	Female (*n* = 56)	Male (*n* = 49)
Female	91.7	66	6	94.6	53	8
Male	87.5	6	42	83.7	3	41
MCC = 0.792				MCC = 0.792		

## Conclusions

Overall, the results of this study demonstrate that Raman analysis of Palmer amaranth leaves can be used to identify the sex of the plants with high accuracy and reliability based on differences in spectral bands associated with nitrate salts, lipids, carotenoids, and terpenoids. These findings provide valuable insights into the sex-specific characteristics of Palmer amaranth and may have implications for the development of sex-specific management strategies for this invasive weed. Further research is warranted to understand the underlying physiological and biochemical mechanisms responsible for the observed spectral differences, and also to investigate the applicability of this technique in small seedlings. Additionally, the highly accurate results of our PLS-DA models also indicate that the spectral differences between male and female plants are significant and can be reliably captured and classified using RS. These results suggest that Raman analysis, in combination with PLS-DA, can be a promising, non-destructive, and efficient method for sex determination of Palmer amaranth plants in field settings, with potential applications for monitoring and managing this invasive weed in real-world environments.

## Author contributions

APH, IJ, SH, and AR performed the experiment, collected, and analyzed spectra. APH and NKG performed PLS-DA; APH, MB, NS and DK conceptualized the idea, supervised the project, wrote the manuscript, and administrated the work.

## Conflicts of interest

There are no conflicts to declare.

## Supplementary Material

RA-014-D3RA06368B-s001
