# Supplementary material for: Elucidation of sex from mature Palmer amaranth (Amaranthus palmeri) leaves using a portable Raman spectrometer
Source: RSC Adv. 2024 Jan 8;14(3):1833–7. doi: 10.1039/d3ra06368b (PMC10772952; doi:10.1039/d3ra06368b)
Supplement: RA-014-D3RA06368B-s001 [file RA-014-D3RA06368B-s001.pdf]

Electronic Supplementary Material (ESI) for RSC Advances.
This journal is © The Royal Society of Chemistry 2024

Elucidation of Sex from Mature Palmer Amaranth (*Amaranthus palmeri*) Leaves Using a Portable Raman Spectrometer

Aidan P. Holman[1,2], Nicolas K. Goff[2,3], Isaac Juarez[1,2], Samantha Higgins[2], Axell Rodriguez[2], Muthukumar Bagavathiannan[4×], Dmitry Kurouski[2,5*], and Nithya Subramanian[4+]

ORCID

Aidan Holman: 0000-0003-4244-7348

Dmitry Kurouski: 0000-0002-6040-4213

1. Department of Entomology, Texas A&M University, College Station, Texas 77843, United States
2. Department of Biochemistry and Biophysics, Texas A&M University, College Station, Texas 77843, United States
3. The University of Texas at Austin Dell Medical School, Austin, Texas 78712, United States
4. Institute for Advancing Health through Agriculture, College Station, Texas, 77843, United States
5. Department of Soil and Crop Sciences, Texas A&M University, College Station, Texas 77843, United States

×Email: muthu@tamu.edu
*Email: dkurouksi@tamu.edu
+Email: Nithya.Subramanian@ag.tamu.edu

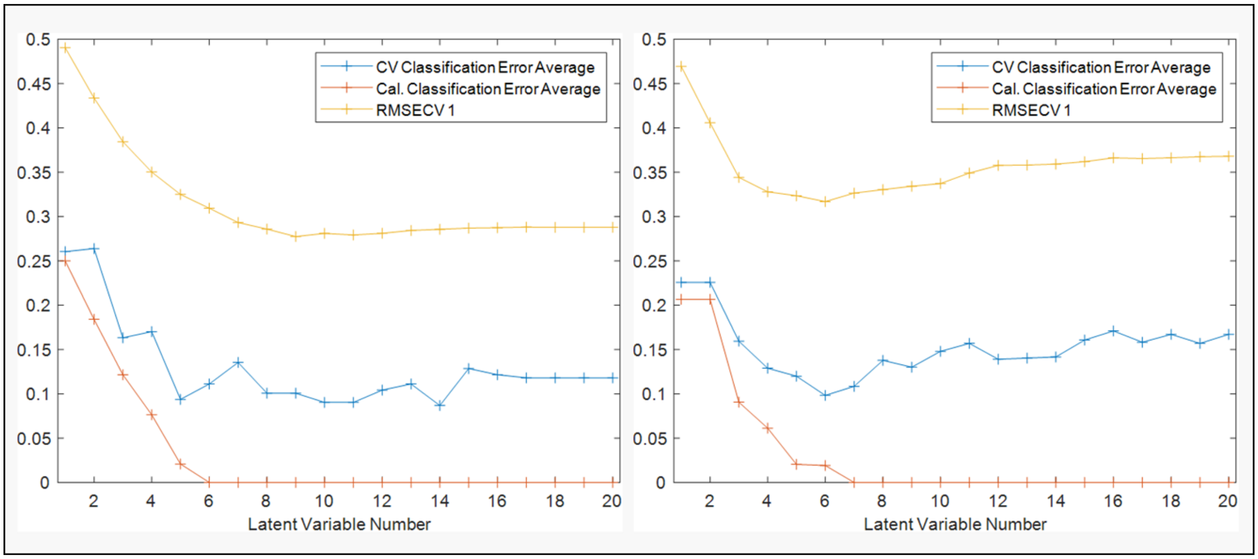

Figure S1. Root-mean-square error of cross-validation (RMSECV) scores and classification error averages from both calibration and cross-validation for PLS-DA of the first run (left) and the second run (right).

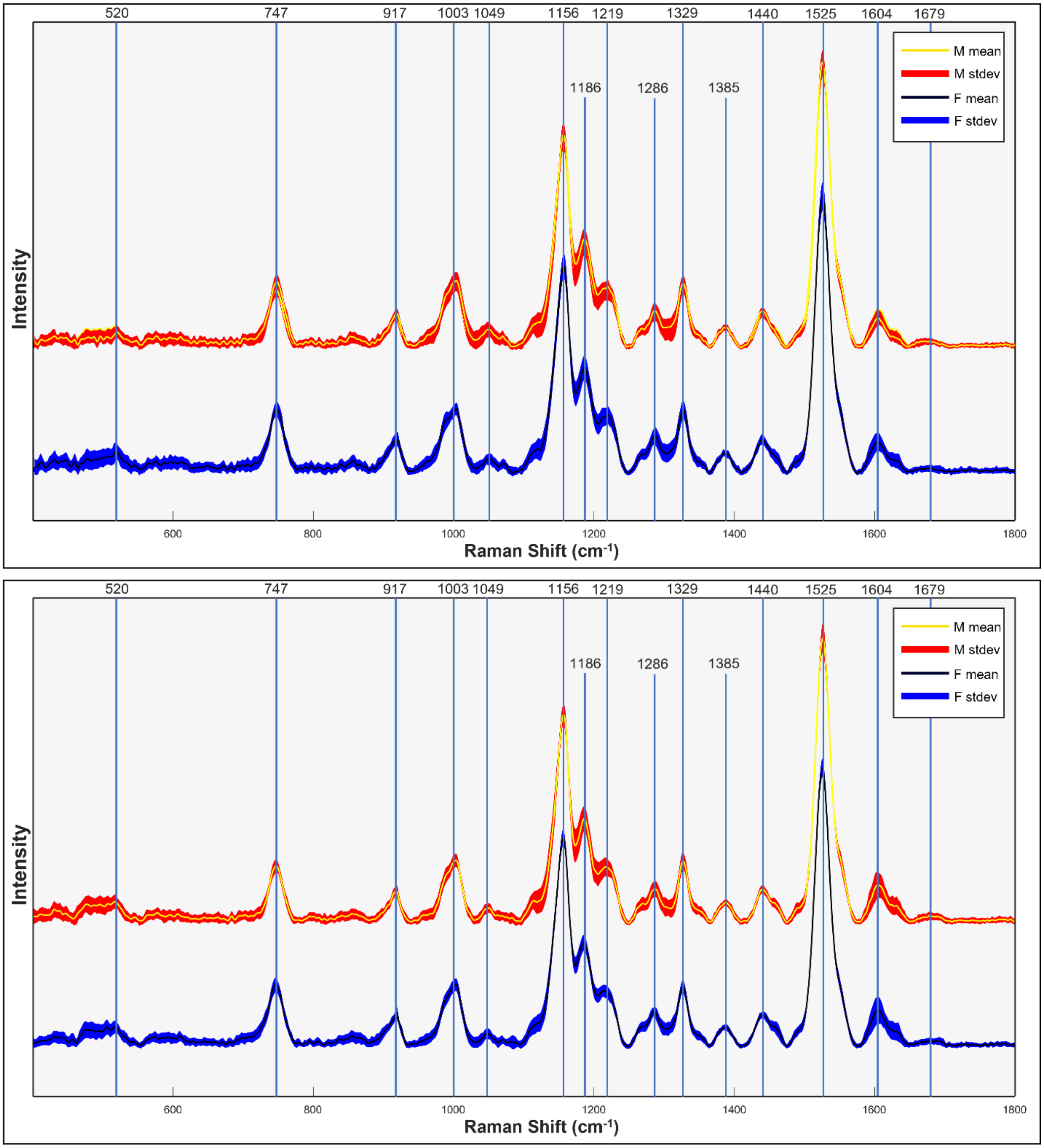

Figure S2. Mean (solid lines) and standard deviations (dashed lines) of Raman spectra of Palmer amaranth leaves, colorized by male (red/yellow) and female (blue/black) for the first run (top) and the second run (bottom).

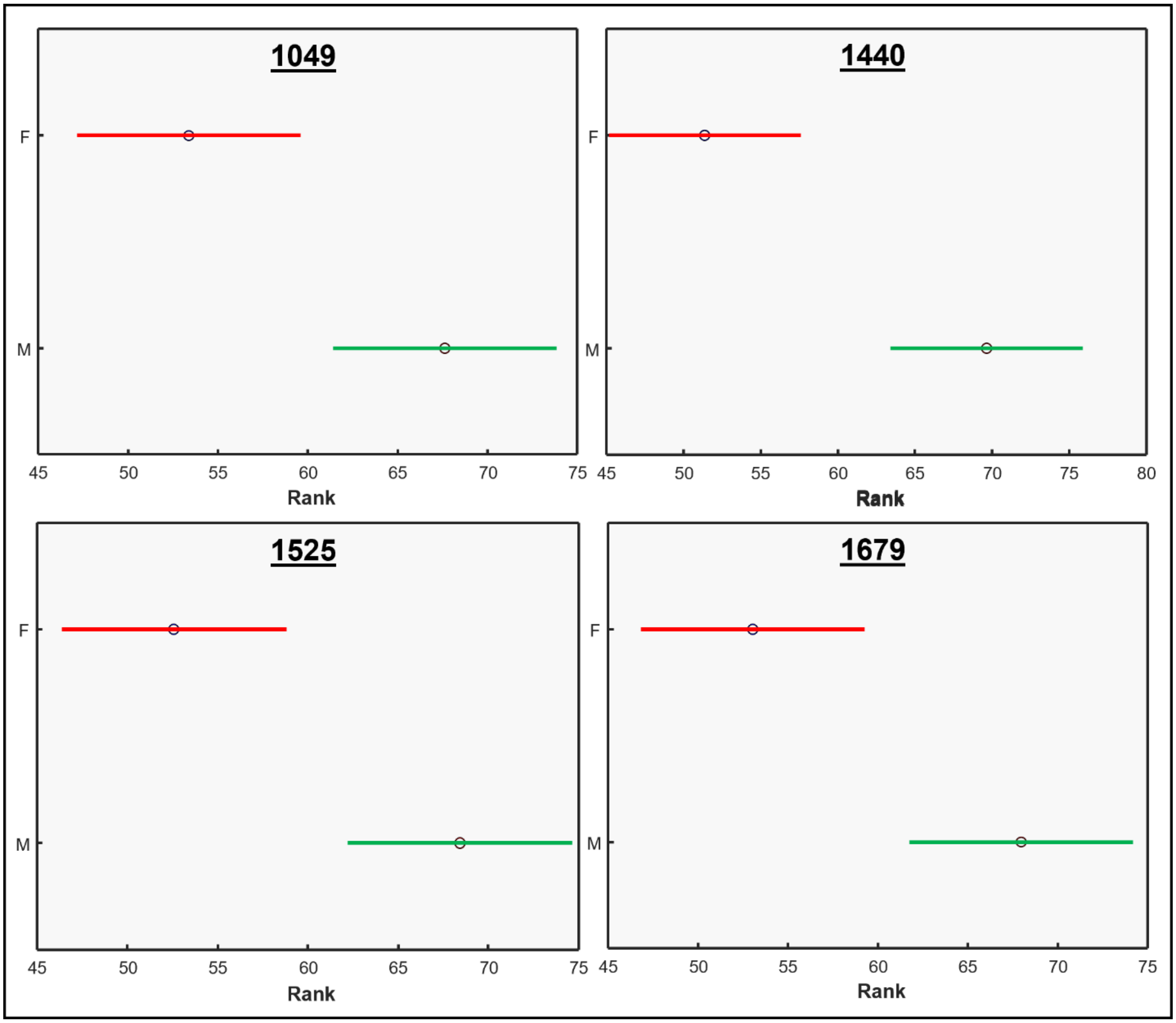

Figure S3. ANOVA tests for bands of physiological importance from the Raman spectra of mature Palmer amaranth leaves, male (green) and female (red). The data displayed is from the second experimental run.
